# Segmentation by motivations in religious tourism: A study of the Christ of Miracles Pilgrimage, Peru

**DOI:** 10.1371/journal.pone.0303762

**Published:** 2024-05-16

**Authors:** Mauricio Carvache-Franco, Otto Regalado-Pezúa, Orly Carvache-Franco, Wilmer Carvache-Franco

**Affiliations:** 1 Universidad Bolivariana del Ecuador, Campus Durán Km 5.5 Vía Durán Yaguachi, Durán, Ecuador; 2 ESAN Graduate School of Business, Universidad ESAN, Surco, Lima, Peru; 3 Universidad Espíritu Santo, Km. 2.5 Vía a Samborondón, Samborondón, Ecuador; 4 Facultad de Ciencias Sociales y Humanísticas, Escuela Superior Politécnica del Litoral, ESPOL, Campus Gustavo Galindo Km 30.5 Vía Perimetral, Guayaquil, Ecuador; National University Bangladesh, BANGLADESH

## Abstract

The present study, focused on pilgrimages as part of religious tourism, aimed to achieve the following objectives: Identify the motivations of the demand for religious tourism focused on pilgrimages; analyze the segmentation of the demand; identify the relationship between demand segments with satisfaction and loyalty; and establish the sociodemographic aspects that characterize demand segments. The study was conducted during the Pilgrimage of the Christ of Miracles in Lima, Peru. The sample was taken on-site from 384 tourists. The statistical techniques used were factor analysis and the k-means clustering method. The results reveal five motivational dimensions: Religious Experience, Belief Experience, Escape, Touristic Experience, and Shopping. Three attendee segments were also identified: Believers, related to belief experience; Religious, related to religious experience; and Passive, tourists with low motivations. The Religious segment had the highest satisfaction and loyalty levels among these groups. Sociodemographic differences were also found in the demand segments. The findings will contribute to management guidelines for destination administrators with religious events and provide insights into academic literature.

## 1. Introduction

The early studies on religious tourism have defined it as tourism that focuses on places of current and past religious significance [1] or as the way people of a specific faith travel to visit important religious sites [2]. Meanwhile, contemporary studies indicate that it is a multifaceted and constantly evolving construct that engages the tourist spiritually, mentally, and emotionally [3,4]. Religious tourism is a form of travel with religious purposes or orientation [5]. Other researchers have stated that the driving force behind religious tourism lies in faith and the connection between the human and the divine, along with the union of deep devotion and spiritual understanding [6,7]. Additionally, the growing search for spiritual fulfillment would increase the demand for religious tourism [8]. In this line, religion is a transcendental force in human travel, especially in pilgrimages [9]. Catholic and Muslim religions have spiritual manifestations in ancient and new places that attract tourists [10]. It is essential to consider that religious tourism provides unprecedented opportunities [11], attracting and motivating a more significant number of tourists despite the secularization and popularization of religions [12].

With this context, the study of religious tourism brings us closer to concepts such as motivation, satisfaction, loyalty, and demand segmentation, which, when interrelated, make it possible for research in this tourism area to continue developing. It is worth adding that, as expressed by several authors, motivation in religious tourism would be exclusively related to religion. It could then be interpreted as a journey of self-transformation for the acquisition of knowledge and status through contact with the sacred [2,13,14]. Within religious tourism, the traveler’s motivation is fundamental for the overall development of tourism [15] as well as tourist satisfaction and loyalty. In this context, satisfaction in religious tourism is defined as the connection of a religious environment with tourists [16]. Loyalty, conversely, is conceived as the intention of loyal tourists to return to and recommend a destination to others [17]. Along these lines, segmentation allows for identifying appropriate target markets that tailor products and services to the needs or preferences of the specific market [18]. This idea is shared by other authors, indicating that destinations that adapt to visitors’ needs would be a decisive factor in their destination choice [19]. Religious tourism, segmentation, and new market niches include retreats, pilgrimages, and spiritual journeys [4].

With these recent ideas, one of the Latin American religious manifestations stands out as a meeting point for the culture and history of the continent. This is important for tourists and the generation of current and future tourist flows in many destinations [20]. We refer to the pilgrimage of "El Señor de los Milagros" or Christ of Miracles in Peru, a Catholic manifestation that began with worshiping the image of Jesus Christ painted in the 17th century. Annually held in October in Lima, it is also replicated in cities such as New York, Rome, and Barcelona, gathering thousands of devotees during its processions. The origin of this pilgrimage dates to a cult of Africans that became a symbol of Peruvian identity [21]. In this context, it is possible that the Andean tradition merged with the worship of the pre-Hispanic God of earthquakes, Pachacamac, whose Sanctuary was the most important on the central coast of Peru [22].

It is important to note that religious tourism in Peru, with its rich amalgam of cultural traditions, spiritual heritage, and pilgrimage destinations, plays a crucial role in the country’s socioeconomic and cultural aspects. The practice, rooted in Peruvian history, not only offers a pathway to the exploration of faith and spirituality but also contributes significantly to the economic development of various regions. As Puccio [23] points out, religious tourism acts as an engine of spatial transformation, generating economic spillover and promoting the development of tourism infrastructure in pilgrimage areas. This interaction between popular religiosity and tourism highlights the capacity of religious practices to mobilize crowds and mark the territory, thus enriching Peru’s cultural and tourism landscape.

Furthermore, international bodies such as UNESCO recognize religious and spiritual aspects as components of intangible heritage and highlight the importance of preserving and promoting these traditions. Martínez [24] highlights how intangible heritage, including religious rituals and festivities, attracts tourists in search of authentic and meaningful experiences, thus contributing to the livelihood of local communities and maintaining cultural identity. In this context, religious tourism emerges not only as a reflection of Peru’s spiritual diversity but also as a sustainable practice that fosters the conservation of cultural heritage and regional economic development, proving to be a permanent practice beyond a fad in contemporary society [25]. It should also be noted that beyond the strictly religious, festivities around religious tourism are presented as a space where the sacred and the profane intertwine, offering moments of deep spiritual connection and opportunities for leisure and cultural exploration. This intertwining invites tourism authorities and managers to develop strategies that improve infrastructure and services and respect and enhance the rich diversity of experiences visitors seek.

For the development of this work, recent literature that has begun to define the religious tourism market based on its motivations, satisfaction, loyalty, and segmentation was analyzed. Since segmentation does not have extensive research on religious tourism, this study provides new insights into this tourism area. To date, religious tourism studies are scarce and no standardized findings on pilgrimages have been found. Understanding the tourist’s motivations and experiences is crucial nowadays to develop strategies in each segment. Since it is necessary to shed light on the segmentation of the religious tourist by motivations to propose destination management plans, the following objectives have been proposed, which will guide the research and contribute significantly to the development of new concepts in pilgrimage-focused religious tourism: (1) Identify the motivations of the demand for religious tourism focused on pilgrimages; (2) Analyze the segmentation of the demand; (3) Identify the relationship between demand segments with satisfaction and loyalty; and (4) Establish the sociodemographic aspects that characterize demand segments.

## 2. Literature review

### 2.1. Motivations for the demand for religious tourism

Motivation is the process that influences the orientation and efforts of an individual to achieve a goal [3,9,26], while tourist motivation reflects the internal needs of tourists, also referred to as push and pull factors [27]. Some authors have indicated that tourist motivations are a multifaceted construct [3] that originates from the intrinsic reasons for the trip and the forces that drive the tourist to choose a particular destination [28]; they are also the specific desires of tourists that lead them to travel [29].

On the other hand, motivation in religious tourism can be considered as the visit to religious places or events by both believers and non-believers [30]. According to Kayal [31], motivations are the most significant driving force in this type of tourism. It should be noted that they have also been referred to as motivational factors that originate from individual commitments to the sacred, which can arise unexpectedly [7]. Other authors have indicated a great diversity of religious motivations among tourists or pilgrims [3,26]. As Belhassen & Bowler [32] pointed out, religious tourism can transform and open up the possibility of seeing things differently. Still, it depends on the tourist to decide what to do with it.

On this basis, tourists are often driven to sacred places by the need to be part of a whole. At the same time, motivations are constructed through intersubjective interactions and the exchange of religious experiences that amplify the authenticity of the places [7]. In this context, Albayrak & Caber [33] explained that religious tourists would like to visit a destination out of curiosity or admiration and benefit from on-site support services, such as excursions and recreational activities. Thus, religious tourism would focus on spirituality and as a significant economic driver attracting substantial capital [4,34]; In this way, belief in a god allows tourists to understand themselves better, connect with like-minded groups, discover new things, and connect spiritually [3,34,35].

In this context, we turn to studies that direct this research toward understanding the diversity of motivations as an essential point to achieve the stated objectives. We highlight the work of Zamani-Farahni & Musa [36], who identified two dimensions of religiosity: belief and Islamic practice. They focused on the Islamic religion and the socio-cultural impact of tourism among various residents of two tourist areas in Iran. Similarly, researchers Scaffidi Abbate & Di Nuovo [37] studied the Sanctuary of Medjugorje in Bosnia and Herzegovina. They found that the travel objectives of both men and women showed a clear difference among religious tourist travelers. Therefore, they established two dimensions: tourist personality and socialization related to friendliness, self-esteem, and cooperation, among others. In this line, a study by Hughes et al. [1] highlighted two types of religious motivation: social motivation (enthusiasts) and religious motivation (experience seekers). They focused their analysis on tourists who visited Canterbury Cathedral in the United Kingdom. Likewise, a study by Lois-González & Santos [38] on the pilgrimage to "Santiago de Compostela" emphasized that this multicultural manifestation unified various motivations to develop a type of tourism they called experiential or spiritual seeking. The authors highlighted religious, cultural, sports, and shopping motivations as the most recurrent among visitors to this route. Other researchers studied the behavior and attitudes of tourists in Hungary at various traditionally religious sites, highlighting various motivations among participants: the need for personal fulfillment, the desire to participate in religious rituals, destination culture, and emotional meaning [39]. similar analysis, based on visits to the sacred relics of Saint Francis Xavier in Goa (India), discovered five motivational factors among pilgrims and site visitors: religious experience, social exploration, escape, belief experience, and shopping [40].

Other motivational factors found at a religious site include religious, tourist, and recreational motivations, which emerged after analyzing the intrinsic values of tourists related to the Sanctuary of Divine Mercy located in Poland [10]. Researcher Dejan Iliev [4] analyzed the transformation of religious tourism from its development and new perspectives to discover various motivational factors in each individual: spiritual and religious; cultural and educational; pilgrimage; health and well-being; search for meaning and purpose; communion and community. Similarly, Robina Ramírez & Fernández Portillo [41] studied motivations in religious tourism from an innovative perspective, basing their study on the Royal Monastery of Guadalupe in Spain. They discovered the importance of education in developing various tourist motivations, including religious, environmental, rural, and cultural motivations. A recent study focused on the religious tourism destination of Mecca [42] established the main motivations of its visitors: religious, social, cultural, and shopping, and then defined a segment of high motivations called "multiple motives." In a study carried out on pilgrimages to Polish sanctuaries, carried out by academics Rosak-Szyrocka et al. [43], they found that the main reason for the visit is related to religious causes (63%); the second for tourist reasons (19%) and the third is related to social reasons (17%). Subsequently, another study on religious places in Saudi Arabia indicated that tourists’ intentions are related to their physiological and self-esteem needs. The author identified six types of motivations: spiritual, cultural and educational, social and communicative, health and well-being, adventure and exploration, and fulfillment of religious obligations [31].

The reviewed literature suggests that motivations in religious tourism have been analyzed from various perspectives. According to the mentioned studies, motivations individually influence tourists, and their intention to visit a religious center is related to their preferences, eventually relating collectively to religious or social contexts. Among the main motivations found, personal, social, and religious aspects stand out, as they drive tourists to participate in a religious event, specifically a pilgrimage. Thus, Our first hypothesis is: H1. In religious tourism there are several motivations, including belief, religious and others related to tourism in general.

### 2.2. Segmentation in religious tourism

Nowadays, market segmentation in tourism seeks to capture the complexity of each experience through regular research [44] without neglecting its core foundations: demographics, geography, behavior, personality, and benefits the tourist seeks [45]. Segmentation is essential for defining the appropriate combination of marketing strategies and tourism promotion [46]. Some studies on tourism segmentation highlight two perspectives: segmentation by motivation and segmentation by benefits [47], which could be based on the personalities and individual needs of each tourist [13,19].

In addition, demand segmentation in tourism research divides the tourist market into groups of tourists with shared perspectives and behaviors [48]. Its objective is to break down the heterogeneous market into small homogeneous groups to understand the demand better and thus rely on selected criteria [19]. It is necessary to add that current literature focused on tourist demand segmentation allows for understanding the complexity of tourists’ experiences and analyzing their previous experiences before a visit [44,48]; intending to attract both religious and non-religious tourists through effective tourism promotion [9] and the development of efficient strategies [17].

It should be noted that the limited literature on demand segmentation in religious tourism shows that tourists’ behaviors are based on positive travel experiences. Among previous findings, researchers Martínez et al. [49] based their study on the destination Santiago de Compostela in Spain. They analyzed tourist satisfaction indicators and segmented two groups of visitors based on their travel motivations: pilgrim tourists and non-pilgrim tourists. Another study used psychographic segmentation to focus on visiting Chester Cathedral (England) tourists. Expectations, perceptions, attitudes, and favorable judgments of tourists about visiting this site were analyzed, and Francis et al. [50] discovered segments of tourists based on visitor type scales. They highlighted two types of tourists: extroverts and introverts. The results suggested that there are eight primary constructs in tourists (extraversion, introversion, sensation, intuition, feeling, perception, judgment, and thinking) associated with personal preferences and their specific expectations. Likewise, Santos et al. [51] analyzed visits to San Miguel Island in the Azores, Portugal, during religious manifestations of Holy Week, where they discovered three types of tourist segments: spiritual, religious, and moderately religious. These categories primarily originated from variables of participants’ attitudes and religious beliefs.

On the other hand, Tkaczynski & Rundle-Thiele [52] segmented tourists interested in observing music bands at music festivals after analyzing attendees’ motivations at the Christian festival Easterfest, held annually in Australia during Easter. This study identified four visitor segments: family-oriented working visitors, local young student visitors, active working campers, and groups of young campers. Similarly, Navruz-Zoda & Navruz-Zoda [53] analyzed the development of religious tourism and pilgrimages to Sufi shrines in Uzbekistan. The authors analyzed motivational variables such as the tourist’s conviction to define two categories: Mumine tourists (people who obey the rules of Islam) and Muslim tourists (considered Muslims who do not trust the rules of Islam but believe in God). This study defined five segments of tourists based on travel motives: Sufi Muslims, non-Sufi Muslims, representatives of other religions, scientific researchers and intellectuals, and other categories of tourists.

Meanwhile, Griffin & Raj [54] analyzed religious tourism and the concept of pilgrimage from a global perspective, collecting data from the most attended pilgrimages on each continent. This work found three groups of tourist segments in the religious context: general and accidental tourists, interested or scholarly tourists, and fervent tourists. According to the authors, this would be the basis for segmenting the tourism market and addressing the needs of each one in the future. In this way, Tkaczynski & Arli [55] identified five segments of people attending conferences and visiting religious islands after participating in the annual conference held in Brisbane, Australia. These segments include first-time attendees from the Baptist group of Sunshine Coast, women under 21 attending the Brisbane group, mixed-confession tourists from Moreton Region, highly motivated ministry men from Brisbane, and older individuals with Western North experience.

A study by Zouni & Digkas [56] based in Thessaloniki, Greece, segmented two groups of tourists with similar characteristics: explorers of religious history and pilgrims. Explorers would be motivated to visit sacred places as a destination, while pilgrims would consider the organization of the journey to a holy place essential. Unlike pilgrims, explorers do not seek to acquire knowledge or get closer to God. In this line, Iliev [4] studied the evolution of religious tourism and determined that new market niches are being created in this area, such as spiritual retreats, New Age trips, volunteer-oriented religious tourism, faith-based cruises, religious conferences, and Christian camps. According to the author, this segmentation process has contributed to the rejuvenation of religious tourism.

On the other hand, Papastathopoulos [46] exemplified this idea by analyzing attachment and place experience in Muslim travelers with similar preferences for tourist services in the United Arab Emirates. This work focused on the religious affiliation of tourists and identified three heterogeneous groups: utilitarian Muslim guests, independent Muslim guests, and leisure-oriented Muslim guests. The most relevant group was utilitarian Muslim guests, which included various societal groups. Another study on Muslim religious tourism on demand segmentation analyzed residents in Bahrain, a country located in the Persian Gulf, and found three demand segments: multiple motives, religious tourists, and passive tourists [42].

With all that has been said, demand segmentation allows understanding of the meanings given by specific information from religious tourists, their motivations, intentions, and expectations within the different groups that can form in each religious event or each visit to a religious center. According to the literature presented in this section, segmentation identifies groups of tourists with similar characteristics, such as the recurring aspects: religious tourists with some religious affiliation, spiritual tourists, and tourists seeking an emotional experience. These common points arise from the preferences of tourists and individuals who engage in religious tourism, hoping to have emotional experiences in religious places. Therefore, this work contributes significantly to the current literature and opens a new research path within tourism. A notable factor in the studies included in this section is the analysis of tourism segmentation based on tourists’ motivations, which would later be related to the satisfaction of tourists visiting a religious center. With all that has been said, this study allows an understanding of the behaviors and preferences of tourists according to their preferences. It lays the groundwork for improving the management of personalized experiences that meet the needs of each tourist by tour operators, who could market their attractions more effectively. Since the literature on pilgrimages is still scarce, our second hypothesis is: H2. There are several segments in religious Tourism, among them, the segment of believers and the religious segment.

### 2.3. Satisfaction and loyalty segmentation in religious tourism

When referring to satisfaction in religious tourism, it can be added that it is related to the mediating effects of awe on tourists [16]. According to Yan & Jia [57], tourists may perceive new prosocial values within a religious environment. Global and attribute-based approaches have been adopted to conceptualize and measure tourist satisfaction [58]. There has been a shift from studying travel motives and tourist satisfaction to exploring the influence of travel experiences and how they can impact subsequent behavior [12]. Several authors indicate that satisfaction with religious tourism is the feeling of awe that positively impacts loyalty [57] and plays a crucial role in the desire to spend money [59].

Furthermore, it is related to motivations regarding the destination [17]. In this sense, Muñoz-Fernández et al. [60] emphasized the importance of measuring tourist satisfaction in tourist destinations as it would allow for evaluating the perception of destination attributes and components. It would also be a relevant antecedent to future behavior or visitor loyalty. In this sense for Żywiołek et al. [61], it is necessary to take care of the complete infrastructure of religious places and meet the needs of pilgrims. Simply offering a place of worship is not enough these days.

Now, loyalty in religious tourism is understood as the willingness or intention of the tourist to return to a religious or sacred destination [31] and would correlate with the results produced by satisfaction. According to various authors, loyalty is the tourists’ intention to revisit the destination and their willingness to recommend it [17,31,62]. Additionally, it would be a significant moderator of place attachment and destination loyalty [14]. In this regard, Kim et al. [5] indicated that those who identify as religious tourists often show notable loyalty to sacred destinations.

On the other hand, loyalty to religious tourism is a positive aspect. According to Martínez et al. [49], it is one of the indicators of satisfaction for the tourist or visitor. These authors based their work on the destination Santiago de Compostela (Spain) and indicated that tourist satisfaction depends on various destination attributes that would determine their future loyalty. In this line, Santos et al. [51] stated that loyalty is the intention of pilgrims to undertake the pilgrimage again or recommend the experience to others. They also defined four loyalty elements in pilgrims: influencing factors, the impact of satisfaction, belonging to a religious community, and management factors in religious tourism. In this line, Wu & Mursid [63] studied the Islamic pilgrimage to Mecca (Saudi Arabia), known as Umrah. They analyzed the factors contributing to the loyalty of tourists visiting this destination. They indicated that interaction with the religious community and cultural experiences in Mecca are fundamental for developing loyalty. This was done by segmenting the data based on similar sociodemographic aspects such as gender, age, and educational level. They concluded that Umrah tourists’ participation influences satisfaction, which, in turn, affects traveler loyalty. In addition to this reference, another study on tourists visiting the city of Mecca focused on the segmentation of religious tourism based on the motivations of those who participated in one of its pilgrimages. The researchers analyzed similar aspects, such as religious beliefs, spiritual experiences, and cultural and social expectations. As a result, it was identified that the Multiple motives and religious segments exhibit the highest satisfaction and loyalty levels [42].

Faced with the limited literature on demand segmentation related to satisfaction and loyalty within religious tourism, a primary research need emerges to expand studies on this theme, which could lead to significant findings soon. Simultaneously, it is essential to understand that the relationship between the two variables aims to address the needs of tourists traveling to a religious center with different expectations and motivations. Therefore, a second need arises for tourism operators worldwide: to implement improvements in the tourism market with offerings tailored to the expectations and needs of each tourist group. Due to the lack of studies on this topic, our third hypothesis: H3. There is a significant positive relationship between the segments with the variables of satisfaction and loyalty in religious tourism.

## 3. Methodology

### 3.1. Study area

The Catholic pilgrimage of the Christ of Miracles is a religious event held annually in October in the historic center of Lima as part of Peru’s popular religiosity. This pilgrimage is known as "Pachacamilla’s Christ" or "Cristo Moreno." Devotion to this procession has extended beyond Lima; it is practiced in approximately 77 cities on all five continents and various regions of Peru.

According to historical evidence, the worship of the Christ of Miracles originated in 1651 when an enslaved Angolan Black person painted the image of a crucified Christ on a wall of his brotherhood’s premises. In 1655, a devastating earthquake shook the city of Lima, destroying infrastructure and causing the loss of numerous lives. However, among the debris, the only image that survived was the painting of the crucified Christ. From that moment on, this place became a space of veneration, where the black and mulatto people from the Pachacamilla area gathered to pray and converse [64].

It is worth mentioning that the first procession took place in 1687, initiating the veneration of the image of this Christ. Initially, it was an event outside the official canons of the Catholic Church; it was part of the cultural resistance of the popular and marginalized sectors of the colonial society in Peru. After several years, the approval of Felipe V was obtained in 1720 and that of Benedict XIII in 1727, allowing them to become the Monastery of Barefoot Carmelites (Nazarenas) and granting them the use of the purple habit. According to historical documents, the Sanctuary would be completed in 1771, and just at the beginning of the 20th century, the worship of the Christ of Miracles gained prominence as members of the Lima aristocratic class began to actively participate in its celebration [65].

Currently, the Sanctuary of the Nazarenes stands as a temple of profound religious significance for Catholicism in Peru. It is located in the Cercado district, in the historic heart of Lima. Behind the main altar, the sacred image of the Christ of Miracles is venerated in its central nave. The pilgrimage routes allow attendees to witness an expression of modern faith that intertwines history with the identity of Peru. Similarly, the procession of the Christ of Miracles is a collective manifestation of faith that involves the participation of devotees and faithful individuals in this religious expression. They accompany the image for long hours during the procession, expressing gratitude and praying to maintain an affectionate connection.

During the pilgrimage, the faithful can consume traditional products such as turrones, anticuchos, rice pudding, purple corn pudding (mazamorra morada), and picarones in nearby streets. Devotees come from different places, both from Metropolitan Lima and the outskirts of Lima, from the country’s interior, and even Peruvians residing abroad who schedule their trips to profess their faith to the Cristo Morado. Due to the Covid-19 pandemic, the pilgrimage was interrupted for three years, resuming its course in October 2022.

### 3.2. Survey, data collection, and analysis

The present study was ethically approved by the ESPOL Polytechnic University of Ecuador. Informed consent was requested in writing and was part of the attached Questionnaire. For this work, initially, research of primary sources and contemporary studies on religious tourism was conducted to find connections with the subject under study. In this process, academic data supporting the research was also reviewed to construct a questionnaire with three sections, designed using available information sources in this area. Additionally, the research team selected other previous studies to build the technical instrument that was applied for data collection.

The questionnaire was composed of 3 sections: The first section, consisting of 8 closed-ended questions (Gender, Marital status, Age, Educational level, Occupation, who accompanied you on the trip, number of times you have attended the procession, level of income per month). It was based on the study by Lee et al. [66]. The second section of the questionnaire was formed from the 16 items presented in the study by [40], which were related to the most prominent motivations in this study. The motivation scale was measured using a 5-point Likert scale (1 not important at all and 5 very important). The Cronbach’s alpha coefficient of the final motivation scale reached a value of 0.74, indicating good consistency among the scale items. The third section of the questionnaire was formed with the satisfaction and loyalty variables found (Overall satisfaction, I have the intention of returning to the procession of El Señor de los Milagros, I intend to recommend the religious event. It was adapted from the study by [67]. In this case, the satisfaction scale includes a 5-point Likert scale (1 disagree and 5 strongly agree).

Similarly, the loyalty scale consisted of 3 items indicating revisit, recommendation, and positive comments from tourists. This scale was developed using a 5-point Likert scale (1 disagree and 5 strongly agree). The questionnaire was designed and validated by a team of experts who analyzed it and made improvements. Subsequently, a pilot test was conducted with a group of 10 people to analyze the questions and their understanding.

The sample included 384 respondents. The sampling was conducted in Lima, Peru, between Tuesday, October 18, and Wednesday, October 19, 2022, during one of the processions of the Christ of Miracles. The sample selection criteria were that the respondents were in the procession of the Christ of Miracles and that they were over 18 years of age. The data collection method was carried out through a convenience sampling method, to the pilgrims who were attending the procession, who were closest to the interviewers and who were willing to participate in the sample.

A sample of 384 individuals was obtained, indicating a margin of error of +/- 5%, a confidence level of 95%, and a 50% variation based on an infinite population to calculate the sample. Subsequently, the surveys were entered into the SPSS Version 26 program for statistical analysis. Among the statistical techniques used was factor analysis, which was utilized to reduce items into smaller factors for a more straightforward interpretation of values. The Varimax rotation method was used to order the factor loadings in high and low values.

The Kaiser method was also employed to determine the number of factors with eigenvalues greater than 1. The Kaiser-Meyer-Olkin (KMO) and Bartlett’s sphericity test were two critical indicators to assess the suitability of the factor analysis model. Another technique used was the k-means clustering method, which helped identify different segments in religious tourism. The Kruskal-Wallis test was used to find differences between the means of segments. The Mann-Whitney U test was used to determine where these differences were found.

### 3.3. Inclusivity in global research

Additional information regarding the ethical, cultural, and scientific considerations specific to inclusivity in global research is included in the Supporting Information (SX Checklist)

## 4. Results

### 4.1. Sample profile

According to the results, 59.1% were women, and 40.9% were men. Regarding marital status, singles and married individuals both accounted for 44%. Meanwhile, 24.5% of the attendees at the religious event were between 31 and 40 years old, followed by 18.8% between 51 and 60. 48.7% of the attendees had a university education, and 12% had postgraduate studies. 24.2% were private employees, while 19% were business people. A high percentage of attendees traveled with their families (59.6%), and 22.4% traveled alone. A significant portion of attendees (44.5%) had attended the religious event more than three times. 34.9% earned between $300 and $600 per month, while 33.1% earned less than $300 per month (see Table 1).

**Table 1:** Sociodemographic variables.

Variable		Percentage
Gender	Male	40.9
	Female	59.1
Marital status	Single	44.0
	Married	44.0
	Other	12.0
Age	Less than 20 years	7.6
	1–30 years old	15.1
	31–40 years old	24.5
	41–50 years old	18.2
	51–60 years old	18.8
	More than 61 years	15.9
Educational level	Primary	2.6
	Secondary	36.7
	University	48.7
	Postgraduate / Master / Ph.D	12.0
Occupation	Student	14.6
	Researcher/Scientist	.3
	Businesspeople	19.0
	Private Employee	24.2
	Public Employee	10.2
	Pensioner	7.8
	Unemployed	6.8
	Other	17.2
Who do you travel with?	Alone	22.9
	With your family	59.6
	With friends	6.3
	With your partner	10.4
	Others	.8
How many times have you attended the procession?	Once	19.3
	Twice	25.3
	Three times	10.9
	More than three times	44.5
What is your monthly income level (Dollars/month)?	Less than $300	33.1
	From $300 to $600	34.9
	From $600 to $900	22.7
	From $900 to $1200	8.1
	More than $1200	1.3

### 4.2. Motivations in religious tourism

A Factor Analysis was used to reduce the items into a smaller number of factors, improving the interpretation of the results. The Varimax rotation method was employed to arrange the factor loadings more clearly. Additionally, the Kaiser method with eigenvalues greater than one was used. The Cronbach’s Alpha for the factors ranged between values of 0.806 and 0.586, indicating a high correlation among the elements constituting each factor. The KMO was 0.724, exceeding the value of 0.7, and Bartlett’s sphericity test was significant, justifying the appropriateness of conducting the factor analysis (see Table 2).

**Table 2:** Motivations in religious tourism.

Variable	Religious Experience	Belief Experience	Escape	Tourist Experience	Shopping
To attend a religious festival	0.819				
To experience a different tradition	0.778				
To share the experience with other believers/pilgrims	0.723				
To visit religious sites (Church of the Nazarenas)	0.693				
To experience the mystery of religion	0.612				
To seek peace		0.792			
To seek spiritual comfort		0.787			
To appreciate/experience the greatness of the purple Christ		0.740			
To appreciate the beauty of the purple Christ		0.708			
To relieve daily stress			0.775		
To escape from routine life			0.753		
To accompany friends or family			0.626		
For vacation				0.856	
To get out of boredom				0.772	
To buy local products					0.877
To buy religious articles					0.804
Cronbach’s alpha	0.806	0.775	0.586	0.655	0.643
Eigenvalues	3.728	2.324	1.905	1.395	1.118
Variance explained (%)	23.297	14.526	11.909	8.718	6.986
Cumulative variance explained (%)	23.297	37.823	49.732	58.450	65.436

According to the values in Table 2, the first dimension was related to the experience of attending a religious event, sharing the experience with other believers, and visiting religious sites. For this reason, it was called Religious Experience. This dimension included 23.30% of the explained variance. The second dimension was named Belief Experience, as it was related to the search for peace and spiritual comfort and the appreciation of the grandeur and beauty of the Purple Christ. This dimension accounted for 14.53% of the explained variance. The third dimension was related to escaping routine and stress and was named escape. This dimension included 11.91% of the explained variance. The fourth dimension was related to vacations, called Tourist Experience. This dimension contained 8.72% of the explained variance. The fifth dimension was associated with purchasing local and religious products and was named shopping. This dimension included 6.97% of the explained variance. These results address our first hypothesis: H1. In religious tourism there are several motivations, including belief, religious and others related to tourism in general.

### 4.3. Demand segmentation in religious tourism

A K-means clustering method has been carried out to analyze the segments according to the motivations for attending the religious event. See Table 3.

**Table 3:** Segmentation in religious tourism.

Variable	Believers	Religious	Passive	Kruskal Wallis	Sig	U de Mann-Whitney
To seek peace	4.6	4.6	4.0	17.370	p<0.05	All except 1–2
To appreciate/experience the greatness of the purple Christ	4.6	4.7	4.1	8.823	p<0.05	All except 1–2
To seek spiritual comfort	4.5	4.5	3.6	26.926	p<0.05	All except 1–2
To appreciate the beauty of the purple Christ	4.5	4.7	3.5	54.463	p<0.05	All
To experience the mystery of religion	3.7	4.4	2.0	154.060	p<0.05	All
To experience a different tradition	3.6	3.8	1.5	136.592	p<0.05	All except 1–2
To attend a religious festival	3.5	4.1	1.7	131.789	p<0.05	All
To visit religious sites (Church of the Nazarenas)	3.6	3.6	2.2	56.857	p<0.05	All except 1–2
To share the experience with other believers/pilgrims	3.3	3.7	2.0	79.635	p<0.05	All
For vacation	2.4	1.3	1.3	54.073	p<0.05	All except 2–3
To accompany friends or family	3.5	2.0	1.6	60.001	p<0.05	All
To escape from routine life	3.0	1.4	1.4	77.521	p<0.05	All except 2–3
To relieve daily stress	2.9	1.3	1.1	92.282	p<0.05	All
To get out of boredom	3.7	1.3	1.5	144.670	p<0.05	All except 2–3
To buy religious articles	1.9	1.8	1.1	36.301	p<0.05	All
To buy local products	1.7	1.5	1.4	9.652	p<0.05	All except 1–2

According to the results, the three segments were well differentiated in their means (significant Kruskal-Wallis test). To determine where these differences were found, the Mann-Whitney U test was used, and it was determined that there were indeed differences in the means. The first segment has been labeled as Believers, with high scores in motivations related to religious belief, such as seeking spiritual comfort and appreciating the grandeur and beauty of the Purple Christ. The second segment was called Religious, with high motivation scores related to the religious experience, such as attending festivals and religious sites. Meanwhile, the third segment was labeled as Passive, with low motivation scores. These results answer our second hypothesis: H2. There are several segments in religious Turism, among them, the segment of believers and the religious segment.

### 4.4. The segments and variables of satisfaction and loyalty in religious tourism

The Pearson Chi-square test has been used to analyze the relationship or association between the segments and the satisfaction variables, intentions to return, and intentions to recommend the religious event. See Table 4.

**Table 4:** Segments and satisfaction and loyalty variables.

Variable	Believers	Religious	Passive	Pearson’s Chi-Square	Sig.
Overall satisfaction	4.81	4.81	4.49	28.756	p<0.05
I intend to revisit the procession	4.59	4.86	4.61	25.835	p<0.05
I plan to recommend my friends to visit procession	4.67	4.92	4.54	38.824	p<0.05

According to the results in Table 4, the Religious segment was the group that had the highest level of satisfaction (4.81), intentions to return (4.86), and recommendations (4.92) compared to the other groups. The Believers segment also had high levels of satisfaction (4.81), intentions to return (4.59), and recommendations (4.67). Therefore, it would be advisable to improve scores related to the belief experience and religious experience to increase the satisfaction and loyalty of attendees to this religious festival. These results address our third hypothesis: H3. There is a significant positive relationship between the segments with the satisfaction and loyalty variables in religious tourism.

### 4.5. Segments and sociodemographic variables

Pearson’s Chi-square coefficient has been used to analyze the relationship or association between the segments and the sociodemographic variables in a religious event. See Table 5.

**Table 5:** Segments and socioeconomic variables.

Variable		Believers	Religious	Passive	Pearson’s Chi-Square	Sig.
Gender	Male	50.8%	35.1%	52.9%	10.234	p<0.05
	Female	49.2%	64.9%	47.1%		
Marital status	Single	46.0%	49.0%	24.3%	19.012	p<0.05
	Married	41.3%	38.2%	67.1%		
	Other	12.7%	12.7%	8.6%		
Age	Less than 20 years	7.9%	8.4%	4.3%	23.424	p<0.05
	21–30 years old	23.8%	15.1%	7.1%		
	31–40 years old	25.4%	24.7%	22.9%		
	41–50 years old	14.3%	21.5%	10.0%		
	51–60 years old	17.5%	15.9%	30.0%		
	More than 61 years	11.1%	14.3%	25.7%		
Educational level	Primary	4.8%	1.2%	5.7%	14.225	p<0.05
	Secondary	34.9%	36.3%	40.0%		
	University	54.0%	47.0%	50.0%		
	Postgraduate / Master / Ph.D	6.3%	15.5%	4.3%		
Occupation	Student	20.6%	15.1%	7.1%	37.968	p<0.05
	Researcher/Scientist	1.6%				
	Businessman	11.1%	17.9%	30.0%		
	Private Employee	33.3%	24.3%	15.7%		
	Public Employee	12.7%	8.0%	15.7%		
	Pensioner	7.9%	6.4%	12.9%		
	Unemployed	4.8%	9.2%			
	Other	7.9%	19.1%	18.6%		
Who do you travel with?	Alone	15.9%	19.1%	42.9%	23.428	p<0.05
	With your family	66.7%	61.0%	48.6%		
	With friends	6.3%	6.4%	5.7%		
	With your partner	9.5%	12.7%	2.9%		
	Others	1.6%	.8%			
How many times have you attended the procession?	Once	20.6%	20.7%	12.9%	31.228	p<0.05
	Twice	34.9%	27.5%	8.6%		
	Three times	7.9%	13.1%	5.7%		
	More than three times	36.5%	38.6%	72.9%		
What is your monthly income level (Dollars/month)?	Less than $300	25.4%	36.3%	28.6%	16.171	p<0.05
	From $300 to $600	46.0%	33.9%	28.6%		
	From $600 to $900	23.8%	21.9%	24.3%		
	From $900 to $1200	4.8%	7.2%	14.3%		
	More than $1200		0.8%	4.3%		

According to the results in Table 5, the first segment, called Believers, consisted of both men and women, singles, and married individuals. A high percentage (49.2%) fell between the ages of 21 and 40. Most were university-educated (54%), with occupations ranging from private employees to students. The majority traveled with their families (66.7%). They had attended the religious event two or three times. A significant percentage (46%) had monthly incomes between $300 and $600. The second segment, Religious, had the highest percentage of women (64.9%) compared to the others. This segment had a high percentage of single people (46%) compared to the other groups, with a substantial portion (46.2%) between the ages of 31 and 50. They were educated, with 47% having completed university and 15.5% having postgraduate studies. Employment status varied, with 24.3% private employees and 17.9% business people. A high percentage traveled with their families (61%) and attended the event twice or thrice. It was the segment with lower incomes; 36.3% had monthly incomes below $300. Meanwhile, the Passive segment had a similar percentage of men and women and the highest percentage of married individuals (67.1%) compared to the other groups. It was also the segment with members over 40 (65.7%). Members of this segment had university (50%) and high school (40%) education levels and had the highest percentage of business people (30%). A significant percentage traveled alone (42.9%). It was the segment that had attended the event the most, with 72.9% having attended more than three times. About 14.3% had monthly incomes between $900 and $1200, making it the segment with higher incomes than the others.

## 5. Discussion

The first objective we set was to identify the motivations of demand in religious tourism focused on pilgrimages. The results show five motivational dimensions for attending pilgrimages as part of religious tourism: Religious Experience, Belief Experience, Escape, Touristic Experience, and Shopping. If we analyze the previous findings related to those of this study, we can establish that the first motivational dimension found is Religious Experience, which was identified similarly by [1,4,38,40–42]. In other words, most studies have agreed that there is a religious motivation for this type of tourism. We have now identified that Belief Experience is also a primary motivation in pilgrimages. The second motivational dimension found in our study is Belief Experience, similarly identified by [36] and [40]. The third motivational dimension found was escape, which was equally found by [40]. The fourth factor we found was Touristic Experience, a dimension similarly identified by Lois-González & Santos [38], who labeled it as cultural. Also, academics Hassan et al. [42] found it social and cultural. That is, this motivational dimension related to tourism had not been previously identified, so people, in addition to visiting cultural places, also engage in tourist activities during religious visits. The fifth motivational dimension identified by our research was shopping, which was found similarly by [38,40,42].

Comparing previous findings with our results, we have contributed to the academic literature by identifying the Touristic Experience dimension, which other authors did not previously identify. Another contribution is finding together five dimensions in pilgrimages that other academics have not found together in the same study. The closest studies are those of Pillai et al. [40] and Hassan et al. [42], which identified four dimensions similar to those found in religious tourism.

As a second objective, our study aimed to analyze the demand segmentation in religious tourism focused on pilgrimages. The results show three segments. The first is the group of Believers, which is related to motivations for belief experience. The second segment is the religious group associated with motivations for religious experience. The third segment is the passive group, with low motivation for religious events. Analyzing previous findings, Martínez et al. [49] found Pilgrims and non-pilgrims very similar to our Believers and Passive but did not find the Religious tourists. Also, Santos et al. [51] found the spiritual and religious groups very similar to our believers and religious groups, but they did not identify our passive tourists. Likewise, Zouni & Digkas [56] identified Pilgrims as identical to our Believers group but did not find the other two groups.

In the same way, academics Hassan et al. [42] found Religious tourists and Passive tourists, but they did not find Believer tourists. Therefore, the findings show that a contribution to academic literature is having identified three segments that other authors had not found in the same religious event for the first time. In other words, this study suggests that, in pilgrimages as part of religious tourism, a group of faithful believers, other religious individuals, and a portion of attendees are passive with low religious motivations and go to the destination with intentions to engage in religious activities as well as social, touristic, and shopping activities.

As the third objective, this study aimed to identify the relationship between demand segments and satisfaction and loyalty in religious tourism focused on pilgrimages. The findings show that the Religious segment consists of tourists with the highest satisfaction and loyalty, making them the ones who influence massive attendance at religious events. Reviewing previous findings, few studies have focused on analyzing this aspect of religious pilgrimages. Among them are the studies of Hassan et al. [42], who identified that multiple religious motives had high satisfaction and loyalty levels in pilgrimages. Our contribution is to have found that Religious tourists have the highest levels of satisfaction and loyalty in variables such as return, recommendation, and speaking positively about pilgrimages as part of religious tourism.

As a fourth objective, our study aimed to establish the sociodemographic aspects that characterize the demand segments of religious tourism focused on pilgrimages. The findings show that Believer tourists include both men and women, singles and married individuals, university-educated, with occupations such as private employees and students, and they traveled with their families.

The Religious segment had a high percentage of women, single people, individuals with university and postgraduate studies, and lower incomes. Different from Hassan et al. [42] where the religious segment was made up mostly of married men, between 21 and 40 years old, with a secondary or university educational level. Passive tourists had a large percentage of married individuals, people over 40 years old, pensioners, business people, higher incomes, and attended religious events more frequently. This significant difference among the various demand segments makes us understand that each segment has different sociodemographic characteristics. Therefore, products should be developed according to this demand to increase their motivation during religious pilgrimage. Finding significant characteristics and differences in sociodemographic aspects is a new contribution to the academic literature on religious tourism.

Regarding the practical implications recommended by this study to enhance motivations for visiting the destination for religious pilgrimage, it is suggested that, concerning the Religious Experience dimension, activities involving worship of the Saint should be implemented, along with events related to religion, inviting church authorities, and adorning the sites through which the pilgrimage passes in homage to the Saint. To improve the Belief Experience dimension, religious acts related to the worship of the Saint could be enhanced, and divine acts should also be linked to sacred scriptures by organizers and church authorities. Additionally, programs with prayers and religious acts among believers could be organized. To enhance the Escape dimension, other recreational events and festivals could be held alongside the pilgrimages to attract tourists. Similarly, to improve the Tourist Experience dimension, visits to surrounding areas and tours to explore the city could be offered. Also, to improve the Shopping factor, sales points for religious souvenirs could be implemented along the pilgrimage route. It is also important to develop strategies to improve the stay of the different segments found in this study. To enhance the stay of Believer tourists, it is necessary to create religious acts during the pilgrimage. To increase the satisfaction and loyalty of Religious tourists, religious activities could be mixed with recreational ones. To increase the attendance of Passive tourists, they could be involved in religious, social, cultural, and tourist activities. In this way, more tourists will attend these religious events, and their repetition in visits will increase. The results of this study will help destination administrators design plans to develop destinations sustainably and assist tourism service providers in developing products in line with the demand found.

## 6. Conclusions

Religious tourism encourages tourists to visit a destination to engage in an activity related to religion and faith. In addition to participating in religious activities, many take advantage of the opportunity to engage in other activities, such as social, cultural, tourist, and shopping. This study set four objectives primarily related to demand segmentation in the context of pilgrimages. This research was conducted during the Pilgrimage of the Christ of Miracles in Lima, Peru. The results reveal significant findings that could be standardized in academic literature. To achieve valid results, motivations of the demand were first established, followed by identifying segments related to satisfaction and loyalty. Subsequently, the sociodemographic characteristics of each identified segment were established.

Five motivational dimensions were identified for attending religious pilgrimages: Religious Experience, Belief Experience, Escape, Touristic Experience, and Shopping. Religious pilgrimages are composed of three attendee segments: the first is the Believers segment, related to motivations for belief experience; the second segment is the Religious, related to motivations for religious experience; the third segment is the Passive, a group with low motivations for religious events. Among these groups, the Religious segment had the highest levels of satisfaction and loyalty, making it the most important segment for developing strategies focused on increasing the satisfaction and loyalty of attendees.

As theoretical implications, five dimensions have been identified in religious tourism focused on pilgrimages. The Touristic Experience dimension, which had not been found in previous findings, has also been identified. Likewise, three demand segments were found that are part of this tourism modality, establishing that the religious tourist group is the most satisfied and loyal to the event. Unique sociodemographic characteristics for each segment were also found, which had not been identified in other previous studies.

As practical implications, the findings will contribute to management guidelines for administrators of religious tourism destinations. Likewise, providers will have a better understanding of their demand and will be able to offer improved products and services. The findings provide differentiated characteristics for each segment that can guide the marketing plans of religious destinations.

Regarding the prospects for religious tourism in Peru, it is essential to highlight the richness of the festive calendar in the country, which integrates celebrations of pre-Hispanic and Hispano-Western roots with trade fairs and events adapted to the demand of modern tourism. It is observed that religious tourism in Peru enjoys a rich culture, history, tradition, and biodiversity that satisfy the different segments of visitors found in the study: Believers, Religious, and Passive, offering them unique and satisfactory experiences for each of the five motivational dimensions found in the study. Therefore, in the practical implication, reference is made to the importance of integrated and synergic management of stakeholders. It is recommended that marketing plans be accompanied by improvements in tourism infrastructure to enrich visitors’ experience of sacred and pilgrimage sites in search of sustainable tourism where the valorization of the country’s cultural and natural diversity is guaranteed.

The main limitation of this study is the application of the convenience sampling method, as tourists who were willing to collaborate and were closer to the surveyors were interviewed. Finally, as a future line of research, it is suggested that segmentation based on the image in religious pilgrimages be investigated. The results of this research contribute to the academic literature and the business.

## Supporting information

S1 Database (XLSX)

S1 File (DOCX)

S2 File (DOCX)
