# Supplementary material for: Segmentation by motivations in religious tourism: A study of the Christ of Miracles Pilgrimage, Peru
Source: PLoS One. 2024 May 16;19(5):e0303762. doi: 10.1371/journal.pone.0303762 (PMC11098431; doi:10.1371/journal.pone.0303762)
Supplement: S2 File — (DOCX) [file pone.0303762.s003.docx]

**SURVEY ON THE MOTIVATIONS AND SEGMENTATION FOR ATTENDING THE PILGRIMAGE OF THE LORD OF MIRACLES (Christ of Pachacamilla)**

The ESPOL University of Ecuador is conducting a study on the motivations and segmentation of religious tourism in the Procession of the Lord of Miracles in Lima, Peru. Your authorization is requested to participate in this research project whose objective is to study religious tourism to develop the destination. Your participation is completely voluntary, if you do not wish to participate there will be no negative consequences. You may withdraw from the study at any time. The response is completely anonymous. There is no associated risk. If you have any questions, you can consult Wilmer Carvache-Franco whose email is: wcarvach@espol.edu.ec

I agree to participate ❑

I don´t agree to participate ❑

**1.-** **Do you live in Metropolitan Lima?**

**1** ❑ Yes **2** ❑ No

**MOTIVATION QUESTIONS**

**2.- Rate from 1 to 5 (1 being a little and 5 being a lot) the reasons for visiting and attending the procession of The Lord of Miracles.**

|  | **Visiting reasons** | **1** | **2** | **3** | **4** | **5** |
| --- | --- | --- | --- | --- | --- | --- |
| 1 | To seek peace |  |  |  |  |  |
| 2 | Appreciate/experience the beauty of the Sanctuary |  |  |  |  |  |
| 3 | To seek spiritual comfort |  |  |  |  |  |
| 4 | To appreciate its architecture |  |  |  |  |  |
| 5 | To experience the mystery of religion |  |  |  |  |  |
| 6 | To attend a religious festival |  |  |  |  |  |
| 7 | For tourism |  |  |  |  |  |
| 8 | To share the experience with other believers |  |  |  |  |  |
| 9 | To satisfy curiosity |  |  |  |  |  |
| 10 | It is an opportunity to get to know Lima and other districts |  |  |  |  |  |
| 11 | For vacation |  |  |  |  |  |
| 12 | To accompany friends or family |  |  |  |  |  |
| 13 | To escape from routine life |  |  |  |  |  |
| 14 | To relieve daily stress |  |  |  |  |  |
| 15 | To get out of boredom |  |  |  |  |  |
| 16 | For religious fulfillment |  |  |  |  |  |
| 17 | To experience a holy atmosphere |  |  |  |  |  |
| 18 | To fulfill a lifelong wish |  |  |  |  |  |
| 19 | To pay respect to the saint's relics |  |  |  |  |  |
| 20 | To redeem/free me from suffering |  |  |  |  |  |
| 21 | To buy religious articles |  |  |  |  |  |
| 22 | To buy local products |  |  |  |  |  |

**3. Please rate from 1 to 5 (1 being a little and 5 being a lot) your overall satisfaction with your visit to The Lord of Miracles procession.**

| **General satisfaction with the visit to the procession** | **1** | **2** | **3** | **4** | **5** |
| --- | --- | --- | --- | --- | --- |
| 1. Overall satisfaction |  |  |  |  |  |

**4. Rate from 1 to 5 (1 being a little and 5 being a lot) your intention to return and recommend a visit to the procession of The Lord of Miracles.**

| **Return** **and recommend the destination variables** | **1** | **2** | **3** | **4** | **5** |
| --- | --- | --- | --- | --- | --- |
| 1. I intend to revisit the procession |  |  |  |  |  |
| 2. I plan to recommend my friends to visit procession |  |  |  |  |  |
| 3. I will say positive things when I talk about the processions after the visit |  |  |  |  |  |

**PERSONAL/SOCIODEMOGRAPHIC QUESTIONS**

**5.- Nationality: 1** ❑ Peruvian **2** ❑ Foreign Country __________________

**6.- Gender: 1** ❑ Male **2** ❑ Female

**7.- Marital status: 1** ❑ Single **2** ❑ Married **3** ❑ Other

**8.- Age: 1** ❑ Less than 20 years old **2** ❑ 21-30 years old **3** ❑ 31-40 years old **4** ❑ 41-50 years old **5** ❑ 51-60 years old **6** ❑ More than 61 years old

**9.- Educational level: 1** ❑ Primary **2** ❑ Secondary **3** ❑ University **4** ❑ Postgraduate / Master / Ph.D

**10.- What is your occupation?**

**1** ❑ Student **2** ❑ Researcher/Scientist **3** ❑ Entrepreneur/businessman **4** ❑ Private Employee **5** ❑ Public Employee **6** ❑ Pensioner  **7** ❑ Unemployed **8** ❑ Other

**11.- Who did you attend The Lord of Miracles procession with?**

**1** ❑ Alone **2** ❑ With family **3** ❑ With friends **4** ❑ With your partner **5** ❑ Other……………

**12.- How many days would you visit the procession of The Lord of Miracles?**

**1** ❑ A day  **4** ❑ 4 days and 3 nights

**2** ❑ 2 days and 1 night **5** ❑ 5 days and 4 nights **3** ❑ 3 days and 2 nights **6** ❑ More than 5 days

**13.- What would be your daily expense per person on your visit to the procession?**

**1** ❑ Less than $50 **4**❑ $150 - $199

**2**❑ $50 -$99 **5**❑ $200 - $249

**3**❑ $100 -$149 **6** ❑ More than $250

**15.- What is your monthly income level (Dollars/month)?**

**1** ❑ Less than 500 USD **2** ❑ From 500 to 1.000 USD **3** ❑ From 1.000 to 1.500 USD **4** ❑ More than 1.500 USD
